# Endolymphatic sac tumor mimicking an aneurysmal bone cyst

**DOI:** 10.1055/s-0044-1789202

**Published:** 2024-08-31

**Authors:** Carlos Dier, Osorio Lopes Abath Neto, Bruno Policeni, Leonardo Furtado Freitas

**Affiliations:** 1 University of Iowa Hospitals and Clinics, Department of Neurology, Iowa City IA, United States.; 2 University of Iowa Hospitals and Clinics, Department of Pathology, Iowa City IA, United States.; 3 University of Iowa Hospitals and Clinics, Department of Radiology, Division of Neuroradiology, Iowa City IA, United States.

**Figure 1:**
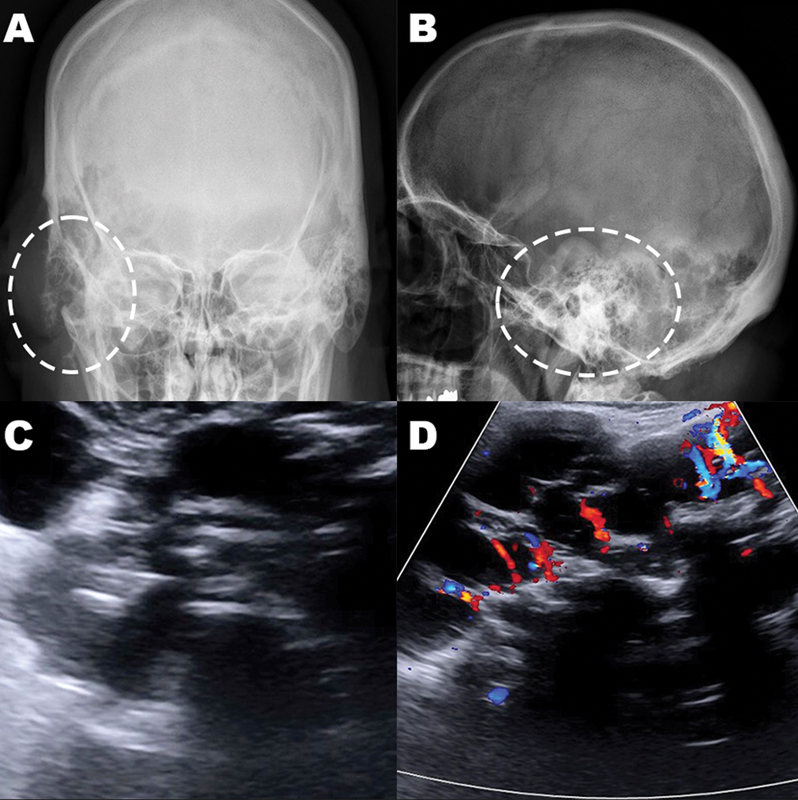
Skull x-ray anteroposterior ( **A** ) and lateral ( **B** ) views showing a large predominantly cystic lesion in the right temporo-occipital region, with soft tissue component and extensive erosion of the mastoid trabeculae (dashed white circles). Superficial soft-tissue grayscale ( **C** ) and doppler ( **D** ) ultrasonography with anechoic content, multiple septations and mild vascularization.

**Figure 2:**
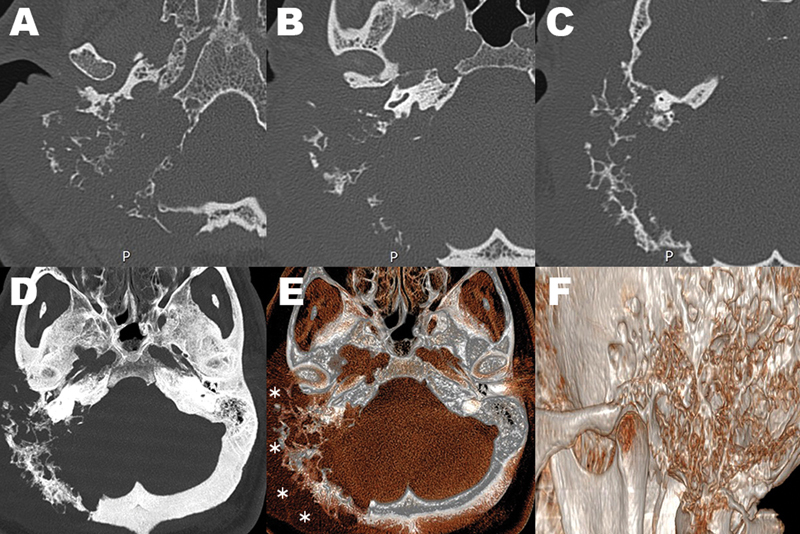
High-resolution computed tomography images of the temporal bones ( **A-F** ). Giant destructive osseous lesion centered in the right mastoid and occipital bones. Multiple spiculated appearance and multicystic soft-tissue component (white asterisks) can be observed.

**Figure 3:**
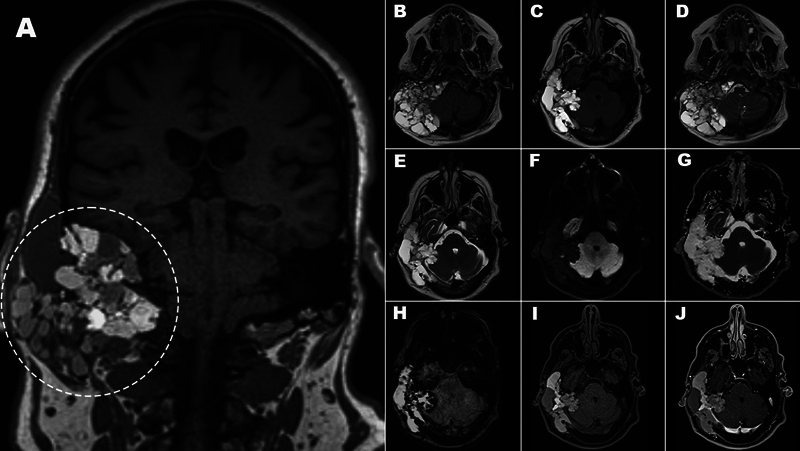
Brain magnetic resonance imaging dedicated to posterior fossa evaluation. Coronal T1-weighted non-contrast ( **A** ), axial fluid-attenuated inversion recovery ( **B-C** ), axial T2-weighted ( **D-E** ), diffusion ( **F** ), apparent diffusion coefficient map ( **G** ), susceptibility-weighted imaging ( **H** ), and fat saturation T1 non-contrast ( **I** ), as well as fat saturation T1-weighted postgadolinium ( **J** ) images. Greater conspicuity of the heterogeneous cystic component, with variable signal due to hyper proteinaceous and hemorrhagic contents. There was facilitated diffusion and no significant enhancement.

**Figure 4:**
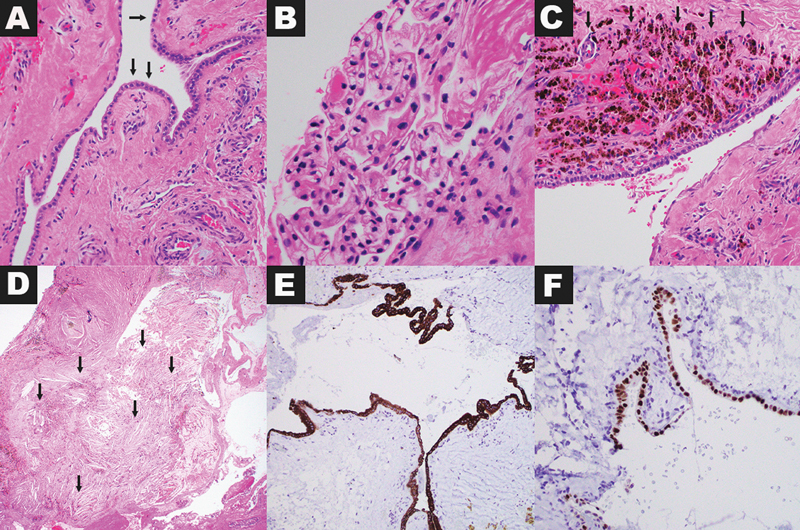
Hematoxylin and eosin-stained slides ( **A and C, 200X; B, 400X, D, 40X** ) show multiple cysts lined by a single layer of epithelium ( **A, arrows** ), focally forming pseudopapillary arrangements ( **B** ). Cysts were associated with extensive hemorrhage and hemosiderin deposition, in many areas undermining the epithelium ( **C, arrows** ). Cholesterol clefts ( **D, arrows** ), morphologic indicators of longstanding tissue reaction, were present in cystic areas and within reactive soft tissue infiltrated by the tumor. Immunohistochemical stains show that the neoplastic cells are positive for pan-keratin ( **C, 100X** ) and PAX8 ( **D, 200X** ).

A 61-year-old female patient presented with a large, right temporal mass causing hearing loss and vertigo. Imaging studies revealed multiple cysts, spiculated lesions, and heterogenous enhancement ( Figures 1 , 2 , 3 ). Soft-tissue components and epithelial cysts ( Figure 4 ) were recognized after surgery.

Endolymphatic sac tumors (ELSTs) are rare adenomatous neoplasms from the vestibular aqueduct's endolymphatic tissue. 1 These tumors are usually associated with Von Hippel Lindau disease. 2 Paragangliomas and hemangiomas should be included in the differential. 3 Endolymphatic sac tumors can mimic the imaging features of aneurysmal bone cysts. 4 Nevertheless, bone spicules and posterior petrous rim expansion on computed tomography and spontaneous hyperintense regions on magnetic resonance imaging suggest ELST. 5
